# Leuco-Prophylaxis in Its Application to Abdominal Surgery

**Published:** 1911-12

**Authors:** 


LEUCO-PROPHYLAXIS IN ITS APPLICATION TO ABDOMINAL SURGERY—O. L. Botero—*La Semana Medica,* Buenos Ayres, August, 1911.

Bottaro uses horse serum, and nucleinic acid 2% for this purpose. The serum is administered subcutaneously in doses of 10 c.c. or applied locally to the peritoneal cavity. The nucleinic acid is used in a similar way.

He has injected 30 c.c. of a 0.50% solution of the acid into the peritoneal cavity per vaginum and cul-de-sac of Douglas. The injections are made 18-24 hours before operation and are followed by no appreciable disturbance.

Acording to his experiments, following the injection of these substances there is a diminution in the number of leucocytes in the general circulation and an enormous increase in the number present in the peritoneal exudate. W.W.Q.

